# 53L10 T-cell engager tribody for immunotherapy of multiple solid tumors

**DOI:** 10.3389/fimmu.2026.1934039

**Published:** 2026-10-06

**Authors:** Margherita Passariello, Martina Belardo, Toshikazu Inoue, Kentaro Kajiwara, Rosa Rapuano Lembo, Shu-ichi Hashimoto, Claudia De Lorenzo

**Affiliations:** 1 Department of Molecular Medicine and Medical Biotechnologies, University of Naples “Federico II”, Naples, Italy; 2 Ceinge-Biotecnologie Avanzate s.c.a.r.l., Naples, Italy; 3 Chiome Bioscience Inc., Tokyo, Japan; 4 European School of Molecular Medicine, University of Milan, Milan, Italy

**Keywords:** anti-cancer mAbs, immune checkpoints inhibitors, immunotherapy, T cell engager antibodies, T cells

## Abstract

Cancer immunotherapy with immune checkpoint inhibitors (ICIs), i.e., antibodies targeting inhibitory ICs, such as PD-1, PD-L1, and CTLA-4, have successfully revolutionized cancer treatment. One of the most promising cancer immunotherapies is based on bi-specific T-cell engagers that simultaneously bind with one arm to a tumor-associated antigen on tumor cells and with the other one to the CD3 complex on T cells to form a TCR-MHC-independent immune synapse. We previously generated four novel tribodies, tri-specific antibody derivative constructs, made up of a Fab targeting 5T4, an oncofetal tumor antigen expressed on several types of tumors, an scFv targeting CD3 on T cells, and an additional scFv specific for an immune checkpoint. In particular, the 53L10 tribody, targeting PD-L1, displayed the best binding properties and stronger anti-tumor activity *in vitro* and *in vivo* in mouse models. Here, we further investigated the anti-tumor potential of 53L10 by testing its effects on a panel of tumor cell types with a more aggressive profile, including gastric, pancreatic, prostate, ovarian, and epidermoid tumor cells. We found that 53L10 was very effective on these cells by inducing stronger T-cell activation and tumor cell lysis than the parental bi-specific 53P lacking an ICI. To further test its therapeutic potential, we also analyzed its stability and the effects on tumor T-cell infiltration *in vivo* on a mouse model. 53L10 was found to be stable after incubation in serum at 37 °C for 3 days and induced a more efficient and earlier increase in tumor tissues of both CD4 and CD8 T cells than the parental bi-specific 53P. These findings support further preclinical evaluation of 53L10 as a new tool for multiple solid tumor-targeted therapeutic approaches.

## 1 Introduction

Immunotherapy based on ICIs (immune checkpoint inhibitors) has gained increasing success in the last two decades. Several monoclonal antibodies (mAbs) targeting immune checkpoints (ICs) are already in clinical use for the treatment of many types of cancer, and the most common examples of checkpoint blockade strategies are those targeting PD-1/PD-L1 and CTLA-4 (1). Some mAbs, such as the anti-PD-1 nivolumab and pembrolizumab or the anti-PD-L1 atezolizumab, have already been approved by the Food and Drug Administration (FDA) for the therapy of melanoma, non-small cell lung cancer (NSCLC), head and neck squamous cell cancer (HNSCC), urothelial cancer, classical Hodgkin lymphoma, and renal cell cancer (2).

However, mAbs binding to single targets still fail in achieving long-term therapeutic response and are effective only in a reduced percentage of patients. Thus, novel approaches led to a switch from standard mAbs to multi-functional molecules that simultaneously hit different targets with the aim of simultaneously “releasing the brakes” and “pushing the accelerator” at the same time (3). In particular, the clinical development of bi-specific antibodies that can act as T-cell engagers, targeting different tumor-associated antigens (TAAs) or ICs, has led to the FDA approval of 11 new drugs in the last 4 years (4). Most of them are T-cell engagers, such as glofitamab (CD20 × CD3), teclistamab (BCMA × CD3), and elranatamab (BCMA × CD3), which, in clinical use, show high overall response rates and efficacy especially for the therapy of lymphoma and multiple myeloma. More recently, even tri-specific molecules containing an additional arm to potentiate the anti-tumor effects are undergoing evaluation in clinical phases. Examples are represented by ISB2001 in phase 1, a tri-specific antibody for relapsed or refractory multiple myeloma, which targets two TAAs on myeloma cells (CD38 and B-cell maturation antigen) and CD3 on T cells (5, NCT05862012); DR30206 in phase 2, which is a tri-specific antibody that simultaneously targets PD-L1, VEGF, and TGF-β in metastatic tumors (6; NCT06132828); and GB268 in phase 1, a tri-specific antibody simultaneously binding PD-L1, CTLA-4, and VEGF (7; NCT06934616).

In this field, we recently designed and constructed novel immunomodulatory bi-specific and tri-specific antibody derivative constructs, starting from the parental bi-specific tribody Tb535H, targeting CD3 on T cells and 5T4 on tumor cells. The novel tri-specific tribodies not only retain the ability of the parental tribody to bind 5T4-TAA and CD3 on T cells, but also can potentially overcome the immunosuppressive environment of solid tumors by an additional scFv derived from an antibody targeting a specific IC, such as anti-PD-L1, anti-PD-1, or anti-LAG-3 mAbs. Among these, the tribody named 53L10, containing the anti-PD-L1 scFv, demonstrated the strongest anti-tumor efficacy *in vitro* and total tumor regression *in vivo* in previous studies on mouse models bearing lung xenografts (8).

Here, we first evaluated the anti-tumor efficacy of 53L10 on a different panel of tumor cells with a more aggressive phenotype, such as gastric, pancreatic, prostate, ovarian, and epidermoid (9) not tested before, and then we investigated its *in vivo* mechanism of action and its stability in physiological conditions.

## 2 Materials and methods

### 2.1 Antibodies

The antibodies used were as follows: the commercial human anti-PD-L1 (MAB0784, G&P Biosciences, Santa Clara, CA, USA), horseradish peroxidase (HRP)-conjugated anti-mouse IgG secondary antibody (62-6520, Invitrogen Thermo Fisher Scientific, Rockford, IL, USA), HRP-conjugated goat anti-human IgG (Fab′)2 mAb (ab87422, Abcam, Biomedical Campus, Cambridge, UK), HRP-conjugated His-Antibody (130-092-785, Miltenyi Biotec, Bergisch Gladbach, Germany), and HRP-conjugated goat anti-Rabbit IgG (H+L) secondary antibody (31460, Invitrogen, Thermo Fisher Scientific).

The human Tb535H, 53L10, and 53P tribodies were generated and produced as previously described (8, 10, 11).

### 2.2 Tumor cell lines

The human tumor cell lines used were as follows: triple-negative breast cancer (TNBC) MDA-MB-231 and BT-549 cells, HER2-negative breast cancer MCF-7 cells, gastric cancer NCI-N87 cells, pancreatic cancer PANC-1 and BxPC-3 cells, ovarian cancer SKOV-3 cells, NSCLC A-549 cells, epidermoid cancer A-431 cells, and prostate cancer DU145 cells, from the American Type Culture Collection (ATCC) (Virginia, USA).

MDA-MB-231 and MCF-7 cells were cultured in Dulbecco’s Modified Eagle’s Medium (11574486, DMEM Gibco, Life Technologies, Paisley, Scotland UK) and Modified Eagle’s Medium (32561029, MEM, Gibco, Life Technologies, Grand Island, USA), respectively. A-549 cells were cultured in Kaighn’s Modification of Ham’s F-12 Medium (10472692, F-12 K Gibco, Life Technologies, Paisley, Scotland UK). NCI-N87, PANC-1, and SKOV-3 cell lines were cultured in Roswell Park Memorial Institute 1640 Medium (12633012, RPMI 1640, Gibco, Life Technologies, Paisley, UK). The media were supplemented with 10% (vol/vol) heat-inactivated fetal bovine serum (50-165-7328, FBS, Sigma-Aldrich, St Louis, MO, USA), 50 U/mL penicillin, 50 μg/mL streptomycin, and 2 mM L-glutamine (all from Gibco Life Technologies, Paisley, Scotland UK).

BxPC-3 cells were cultured in RPMI 1640 Medium (A1049101, ATCC modified, Thermo Fisher Scientifc). A431 cells were cultured in DMEM (08458045, Nacalai Tesque, Kyoto, Japan), and DU145 cells were cultured in MEM (21443-15, Nacalai Tesque, Kyoto, Japan). The media were supplemented with 10% (v/v) FBS (SH30088.03, Hyclone, Cytiva), 100 U/mL penicillin, and 100 μg/mL streptomycin (Nacalai Tesque, Kyoto, Japan). Mycoplasma contamination was assessed using the MycoAlert™ Mycoplasma Detection Kit (Lonza, LT07-218) according to the manufacturer’s instructions.

Human peripheral blood mononuclear cells (hPBMCs) were purchased from ATCC (PCS-800-011). All tissues used for isolation by ATCC conform to the privacy rule guidance and were obtained under informed consent and conform to HIPAA regulations to protect the privacy of the donor’s Personally Identifiable Information. hPBMCs used in the experiments were derived from five different donors.

hPBMCs were thawed out by using a medium composed of RPMI 1640 with 1% L-glutamine, 1% CTLWash™ (CTLW-010, Immunospot by Cellular Technology Limited, Shaker Heights, OH, USA), and 100 U/mL Benzonase (K501 45697 818, Merck Millipore, Darmstadt, Germany) and cultured at 37 °C in R10 medium (RPMI 1640 supplemented with 10% inactivated FBS, 1% L-glutamine, 50 U/mL penicillin, 50 μg/mL streptomycin, and 1% HEPES; 15630-056, Gibco, Life Technologies).

### 2.3 ELISAs

Enzyme-linked immunosorbent assays (ELISAs) were performed to analyze the functional binding of 53L10, after incubation in 100% FBS at 37 °C for 24, 48, and 72 h. Human PD-L1/Fc recombinant protein was immobilized at a concentration of 5 μg/mL in a Nunc 96-well plate for 72 h at 4 °C. The plate was blocked with phosphate-buffered saline (PBS)/milk 5% for 1 h at 37 °C. The untreated or treated tribody 53L10 was incubated at concentrations of 30 and 100 nM for 1 h 30 min at room temperature (RT). FBS or anti-PD-L1 commercial Ab was used as negative or positive control, respectively. After extensive washes, an HRP-conjugated anti-His tag antibody was added for 1 h at RT. The detection was carried out by incubating with 3,3′,5,5′-tetramethylbenzidine (T8665, TMB, Sigma-Aldrich), the reaction was stopped with 1 N HCl, and absorbance was measured at 450 nm with an Envision plate reader (PerkinElmer, San Diego, CA, USA), as previously described (12).

Cell ELISAs were performed to check the expression levels of 5T4 and PD-L1 on breast MDA-MB-231 and MCF-7, lung A-549, gastric NCI-N87, pancreatic PANC-1, and ovarian SKOV-3 cancer cell by using the anti-PD-L1 commercial Ab or the parental bivalent anti-5T4 tribody at a concentration of 50 nM. Tumor cells were plated in triplicate into round-bottom 96-well plates (2 × 10^5^ cells/well), blocked with PBS/BSA 6% for 30 min, and then incubated with the antibodies in PBS/BSA 3% for 1 h 30 min at RT under gentle agitation. After extensive washes, the secondary goat anti-human IgG (Fab′)2 mAb and anti-human HRP-conjugated IgG (Fab′), respectively, were added for 1 h, and detection was carried out by reading the absorbance at 450 nm, as previously described (13).

### 2.4 LDH cytotoxic assays and morphology analyses

To test the cytotoxic effects of 53L10, target tumor cells were co-cultured with hPBMCs [effector-to-target cell (E/T) ratio 5:1] in 96-well flat-bottom plates and incubated for 48 h at 37 °C in the presence of 53L10 used at increasing concentrations (0.1–1 nM). After treatments, the supernatants of the co-cultures were collected by centrifugation at 1,200 rpm for 10 min and the supernatant was tested for LDH release in co-cultures by using the LDH Detection Kit (C20300, Thermo Fisher Scientific). Tumor cell lysis was analyzed by measuring the fold increase of LDH in the presence of each treatment, with respect to the amount present in the supernatant of untreated co-cultures (spontaneous LDH activity) and expressed as percentage with respect to the maximum lysis represented by the cells treated with 10% Triton X-100 as previously described (14). The formula used for calculating the % of cytotoxicity is the following:


$$Cytotoxicity\left(\%\right)\frac{\left(Compound-treated\;LDH\;activity-Spontaneous\;LDH\;activity\right)}{\left(Maximum\;LDH\;activity-Spontaneous\;LDH\;activity\right)}\times 100$$

In parallel assays, spontaneous effector cell release, represented by hPBMCs cultured alone, and spontaneous target cell release, represented by tumor cells alone, were measured as well.

Images of NCI-N87, PANC-1, and SKOV-3 cells, untreated or treated with 53L10 in the presence of hPBMCs, were acquired and processed by using digital microscopy from Zen 3.2 Cell Discover. Cells co-cultured with hPBMCs and treated with an unrelated human IgG1 were used as negative control.

The cytotoxic activity of 53L10 was also evaluated in co-cultures of various cancer cell lines or CHO-K1 cell lines expressing varying levels of 5T4 or PD-L1 and hPBMCs pre-activated with anti-CD3/anti-CD28 beads. Cells were mixed at an E/T ratio of 5:1 or 10:1. The tribody 53L10 was added at a final concentration of 10^5^ to 10^−1^ pM for 72 h. The LDH released in the culture supernatant was measured with LDH-Glo™ Cytotoxicity Assay (J2380, Promega, Milano, Italy) following the manufacturer’s instructions.

### 2.5 Cytokine secretion assays

To evaluate the effects of the antibodies on immune cell activation, the secretion of human IL-2 and IFNγ in supernatants of co-cultures of tumor cells with hPBMCs was assessed by using the DuoSet ELISA kit assays (DY007B, Bio-Thecne R&D Systems, Inc., Northeast, Minneapolis, USA). The culture supernatants were collected after treatments and the cytokines were quantified by using the IL-2 (DY202) and IFNγ (DY285B) kits, according to the manufacturer’s recommendations. Absorbance values were converted in pg/mL by plotting the mean absorbance for each sample in comparison with the standard curve constructed within the experiment.

### 2.6 TIL analysis of PBMC-humanized xenograft model

hPBMCs from healthy donors (CTL Laboratories, ShenZhen China) cultured in ImmunoCult-XF T Cell Expansion Medium (10981, STEMCELL Technologies, Vancouver, Canada) in the presence of 30 U/mL of recombinant hIL-2 (200-02-50UG, Thermo Fisher Scientific) were activated by Dynabeads Human T-Activator CD3/CD28 (11131D, Thermo Fisher Scientific) for 3 days. After removing magnetic beads, hPBMCs were further cultured in the medium containing 30 U/mL of rhIL-2, and then used for transplantation.

A-549 tumor cells (5 × 10^6^ cells) (ATCC) were transplanted into the right flank of 5-week-old female NOD.CB17-Prkdcscid/J mice (Jackson Laboratories Japan, Bar Harbor, ME). On the day the average tumor volume reached 100 mm³, mice were treated with anti-asialo GM1 pAb (014-09801, Fuji Film Wako, Osaka, Japan). In the following day (day 0), 3 × 10^7^ activated hPBMCs were introduced intraperitoneally, and doses of the tribodies were subsequently administered intravenously once every 2 days. Mice were sacrificed and tumors were resected on days 6 and 10 for analyses of T-cell infiltration.

Tumor tissues were harvested from three mice per group and pooled for analysis. Tumor tissues were mechanically and enzymatically dissociated into single-cell suspensions using the gentleMACS Octo Dissociator and the Tumor Dissociation Kit (130-095-929, Miltenyi Biotec) according to the manufacturer’s instructions. Briefly, tumor samples were minced into small fragments and processed using the gentleMACS program with enzyme mixtures provided by the kit. The resulting cell suspensions were filtered through a 100-µm cell strainer to remove debris and washed with cold PBS containing 1% FBS.

Cells were then counted and washed twice with cold PBS containing 1% FBS. For surface staining, cells (1.6 × 10^6^ cells per sample) were incubated with Human Trustain FcX (422305, BioLegend, San Diego, CA, USA) and mAbs specific for CD45 (clone 2D1, FITC-conjugated, 368508, BioLegend; 1 μg/mL), CD4 (clone RPA-T4, Brilliant Violet 605-conjugated, 562658, BD Biosciences; 1 μg/mL), and CD8 (clone SK1, PerCP/Cyanine5.5-conjugated, 344710 BioLegend; 0.5 μg/mL) in Brilliant Stain Buffer (563794, BD Biosciences) for 30 min at 4 °C in the dark. After staining, cells were washed twice with PBS containing 1% FBS and resuspended in PBS containing 1% FBS for analysis.

Appropriate isotype and unstained controls were included for gating. Dead cells were excluded by using Fixable Viability Stain 780 (BD Biosciences).

Data acquisition was performed using a flow cytometer FACSLyric (BD Biosciences, San Jose, CA, USA), and at least 300,000 events were collected per sample. Data were analyzed using FlowJo software (version 10.10, BD Biosciences). Gating was performed sequentially to exclude debris based on forward scatter (FSC) and side scatter (SSC), followed by selection of single cells using FSC-A versus FSC-H. Live cells were then gated by excluding Fixable Viability Stain 780-positive dead cells. Among live cells, CD45^+^ leukocytes were selected, and subsequent analysis of CD4^+^ and CD8^+^ T-cell populations was performed.

### 2.7 Quantitative flow cytometry analysis

Cell surface antigen density was quantified by quantitative flow cytometry using QIFIKIT^®^ (K007811-8; Agilent Technologies, Santa Clara, CA, USA) according to the manufacturer’s instructions. Anti-human 5T4 mouse mAb (Cat. No. MAB49751; R&D Systems, Minneapolis, MN, USA) and anti-human PD-L1 mouse mAb (clone MIH1, purified NA/LE; Cat. No. 565959; BD Pharmingen, BD Biosciences, San Jose, CA, USA) were used as antigen-specific antibodies, and mouse IgG1 isotype control antibody (clone 2E12; Code No. M075-3; Medical & Biological Laboratories, Nagoya, Japan) was used as isotype control antibody. Expression values were calculated by subtracting the ABC value obtained with the negative control antibody from that obtained with the antigen-specific antibody.

### 2.8 Stability analyses

To test the integrity and stability of 53L10 after incubation in serum for increasing time intervals at 37 °C, aliquots were collected after 24, 48, and 72 h and were analyzed by Western blotting under non-reducing conditions. The nitrocellulose membrane was blocked with 0.1% tween-PBS/milk 5% for 1 h at 37 °C and then incubated with an HRP-conjugated anti-His antibody for 1 h at RT. The signals were detected by using a ChemiDoc imaging system (Bio-Rad), and the intensity of the bands was visualized by using Image Lab software (version 6.0.1).

### 2.9 Statistical analyses

Error bars were calculated by considering the results obtained by at least three determinations obtained in three independent experiments. Error bars depict means ± SD. Statistical analyses were assessed by Student’s *t*-test for comparisons between two groups, or one-way analysis of variance (ANOVA) followed by Tukey’s *post-hoc* test for comparisons among three or more groups, based on data from at least three independent biological experiments. Statistical significance was established as ***p ≤ 0.001; **p < 0.01; *p < 0.05, as reported in **Figure 1**.

**Figure 1:**
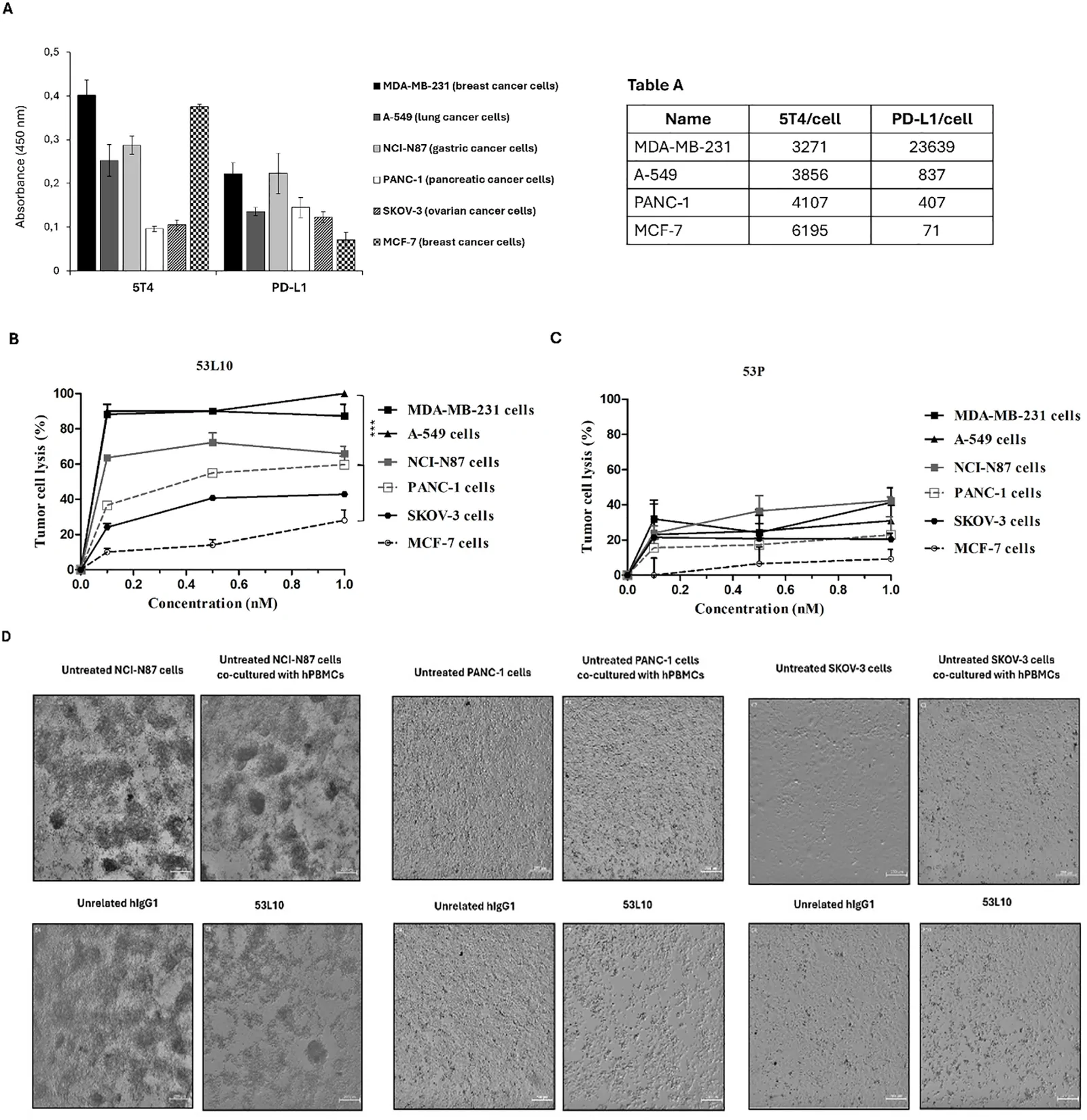
Expression of 5T4 and PD-L1 and anti-tumor effects of 53L10 on a panel of tumor cells. **(A)** The expression levels of 5T4 and PD-L1 on breast MDA-MB-231, lung A-549, gastric NCI-N87, pancreatic PANC-1, and ovarian SKOV-3 cancer cells were tested by cell ELISAs by using a commercial anti-PD-L1 or anti-5T4 antibody followed by their respective HRP-conjugated secondary Abs. The breast MCF-7 cell line was used as negative control for PD-L1 expression. Binding values were reported as the mean of determinations obtained in three independent biological experiments. Error bars depicted means ± SD (Table A). The table reports the expression levels (number of cell surface 5T4 and PD-L1) obtained by quantitative flow cytometry by QIFIKIT (Dako) analyses on MDA-MB-231, A-549, PANC-1, and MCF-7 cells. **(B, C)** Tumor cells were co-cultured with hPBMCs (5:1 effector-to-target cell ratio) and treated with 53L10 **(B)** or 53P **(C)** used at increasing concentrations for 48 h. The effects on tumor cell lysis were reported as dose–response curves. As a negative control, MCF-7 cells were tested. **(D)** Images of NCI-N87, PANC-1, and SKOV-3 cells untreated or treated with 53L10 in the presence of hPBMCs were acquired and processed by using a digital microscopy from Zen 3.2 cell discover. Cells co-cultured with hPBMCs and treated with an unrelated human IgG1 were used as a negative control. Scale bar, 200 μm.

## 3 Results

### 3.1 Effects of 53L10 on a panel of aggressive types of tumor cells

Novel tri-specific T-cell engagers containing three different binding arms, i.e., the anti-TAA, anti-CD3, and anti-IC moieties, were recently generated in our laboratory, and one of them, named 53L10, including an anti-5T4 Fab and two scFvs specific for CD3 and PD-L1, has already shown the most promising anti-tumor efficacy *in vitro* and complete tumor regression *in vivo* (8).

Here, to expand the potential tumor target cells sensitive to 53L10 for therapeutic applications, we further investigated its anti-tumor efficacy by using a new panel of tumor cells, including gastric, pancreatic, and ovarian not tested before. In parallel assays, we used lung (A-549) and breast cancer (MDA-MB-231 and MCF-7) cell lines, previously reported in Passariello et al. (8) only as control references to compare the efficacy of 53L10 on all the tumor cell lines.

Firstly, we performed cell ELISAs to check the expression levels of 5T4 and PD-L1 on the following new cell lines: NCI-N87 from gastric cancer, PANC-1 from pancreatic carcinoma, and SKOV-3 from ovarian cancer. In parallel assays, MDA-MB-231, A-549 (5T4^+^/PD-L1^+^), and MCF-7 (5T4^+^/PD-L1^-^) cells, previously tested (8), were used as controls.

All the cell lines were found to express the targets at detectable levels by cell ELISA (**Figure 1A**), and quantitative flow cytometry analyses on some of these cell lines confirmed comparable levels of 5T4 and higher expression of PD-L1 on MDA-MB-231, A-549, and PANC-1, whereas much lower levels of PD-L1 were found on MCF-7 cells (see Table A in **Figure 1**). Once the presence of the two targets was confirmed, all the cells were incubated with increasing concentrations (0.1–1 nM) of the 53L10 tribody to investigate its cytotoxic effects. In detail, the cells were plated in 96-well plates, in the absence or in the presence of human lymphocytes (5:1 E/T ratio) and treated with the tribody at 37 °C for 48 h under the same conditions previously reported (8) to better compare the results. In parallel, untreated cells or cells treated with an unrelated hIgG_1_ were used as negative control; after the treatments, the supernatants were collected to measure the levels of LDH released as a marker of tumor cell lysis. Dose–response curves of tumor cell lysis are reported in **Figure 1B**, and they positively correlated with the expression levels of 5T4 and PD-L1. In particular, the anti-tumor effect was found to be stronger on the gastric NCI-N87 and pancreatic PANC-1 cells expressing high levels of 5T4 and PD-L1, reaching 60% and 70% of lysis, respectively; a smaller effect corresponding to approximately 50% of lysis was observed on the ovarian SKOV-3 cells expressing lower levels of the two targets. No effects were observed on MCF-7 cells not expressing PD-L1 and used as a negative control. These findings suggest that cell lines expressing similar levels of 5T4 show higher sensitivity to 53-L10 when they express higher levels of PD-L1.

A further analysis of the clusters of reactive lymphocytes shown in the images acquired and processed by using a digital microscopy (**Figure 1D**) confirmed that the activation of lymphocytes against cancer cells was more evident in NCI-N87 and PANC-1 cells. In parallel, the effects of the parental bi-specific tribody, named 53P, which is made up of a Fab domain specific for 5T4, an scFv targeting CD3, and an additional unrelated scFv, but lacking the anti-IC domain differently from the tri-specific 53L10, were tested on the same tumor cells. As shown in **Figure 1C**, an average tumor cell lysis of 20%–40% was measured for the parental bi-specific tribody, which was much lower than the potent effects (60%–70%) observed with the tri-specific 53L10. To further demonstrate the activation of lymphocytes, the release of pro-inflammatory cytokines was measured by ELISA and reported in **Supplementary Figure S1**. The levels of secreted IL-2 and IFNγ were much higher in the supernatant of co-cultures treated with 53L10 than those induced by the treatment with 53P.

To further investigate the anti-tumor efficacy of 53L10, its effects were also evaluated in co-cultures of other breast, prostate, pancreatic, and epidermoid carcinoma cell lines with hPBMCs pre-activated with anti-CD3/anti-CD28 beads (E/T ratio of 5:1 or 10:1). The co-cultures of A431 cells from epidermoid carcinoma, BxPC-3 from pancreatic carcinoma, DU145 from prostate carcinoma, and BT549 triple-negative breast carcinoma cells were treated with the tribody used at lower concentrations (pM range) for 72 h and then the release of LDH in the culture supernatants was measured. As shown in **Figure 2A**, 53L10 was very effective on all the cell lines tested, showing EC_50_ values in the pM range, even though a clear correlation between the expression of targets measured by using quantitative flow cytometry by QIFIKIT (Dako) and EC_50_ was not confirmed on these cells (**Table 1**).

**Figure 2:**
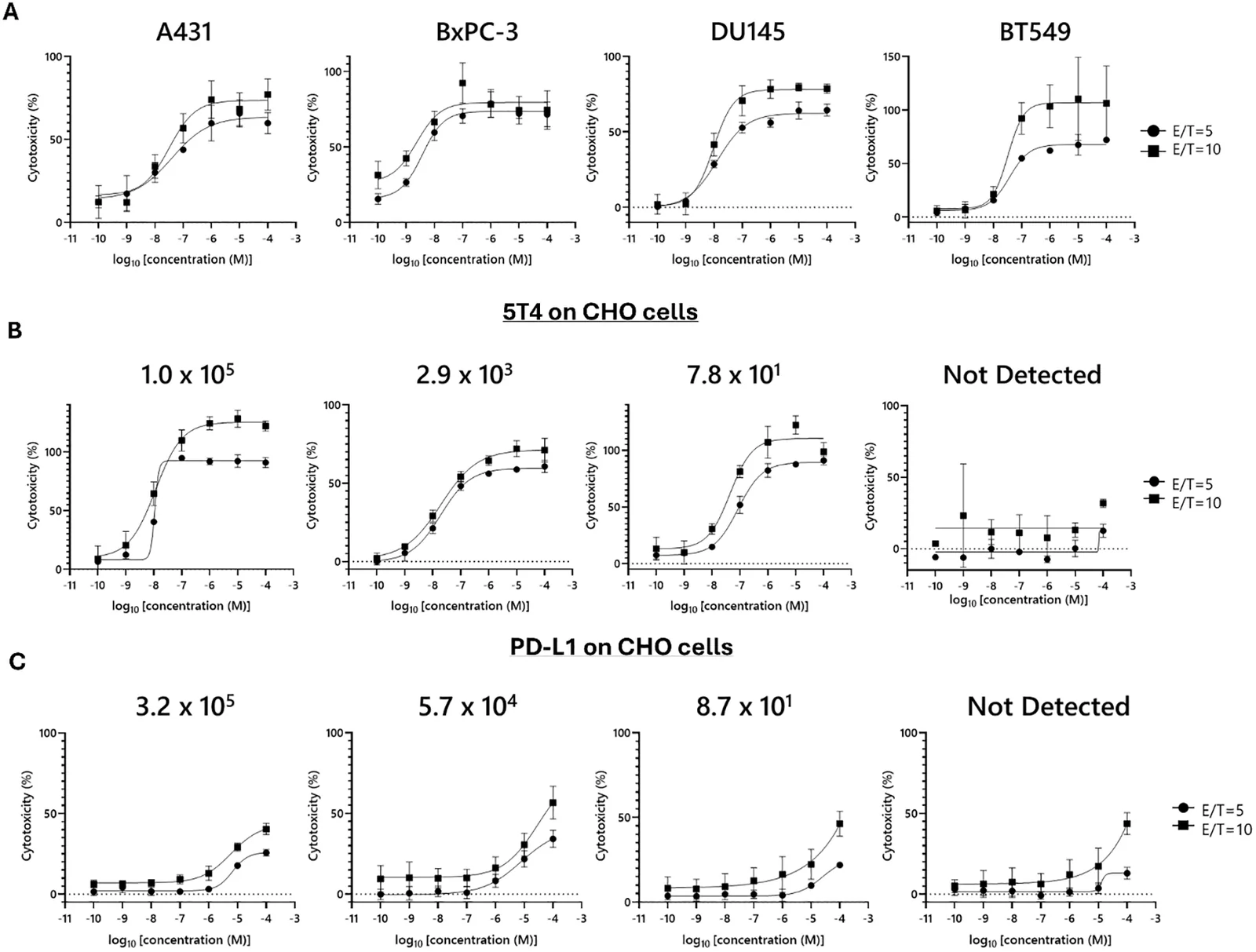
Cytotoxicity and target expression levels on other breast, prostate, pancreatic, epidermoid carcinoma cell lines and CHO-transfected cells. **(A)** The cytotoxic activity of 53L10 was evaluated in co-cultures of various cancer cell lines and human PBMCs pre-activated with anti-CD3/anti-CD28 beads. Cells were mixed at an effector-to-target cell ratio (E/T ratio) of 5:1 or 10:1. 53L10 was added to achieve a final concentration of 10^−4^ to 10^−10^ M. After 3 days of culture, the amount of LDH in the culture supernatant was measured with LDH-Glo™ Cytotoxicity Assay (Promega) following the manufacturer’s instructions. Measures were performed in triplicates at each point. Cytotoxicity percentage was calculated according to the following formula:

$$Cytotoxicity\left(\%\right)=\frac{\left(Effector\;target\;cell\;mix-Effector\;cell\;control-Low\;control\right)}{High\;control-Low\;control}\times 100$$

**(B, C)** Cytotoxic effects of 53L10 on CHO-K1 cells stably transfected with DNA encoding 5T4 or PD-L1 and expressing different levels of the targets. CHO-K1 cell lines expressing varying levels of 5T4 or PD-L1 were established and subjected to a cytotoxicity assay in the presence of 53L10 and human PBMCs pre-activated with anti-CD3/anti-CD28 beads. LDH release was evaluated after 3 days of culture. The cytotoxic activity of 53L10 against CHO-K1 cells expressing human 5T4 or human PD-L1 is shown in **(B)** and **(C)**, respectively. The number of cell surface-expressed human 5T4 or human PD-L1 was determined using the QIFIKIT (Dako) and is indicated above each graph.

**Table 1:** Cell surface expression of 5T4 and PD-L1 and EC50 values for 53L10 effect on tumor cells. The values were calculated by fitting the dose–response curves reported in **Figure 2** after treatment of the cells with hPBMCs from three different donors and performed in triplicates.

Name	5T4/cell	PD-L1/cell	EC_50_ (×10^−9^ M) E/T = 5	EC50 (×10^−9^ M) E/T = 10
A-431	12,633	22,262	32.32	22.62
BxPC-3	4,953	8,721	3.431	3.333
DU145	5,584	8,718	12.07	9.41
BT-549	3,341	4,902	35.83	31.03

To better investigate the specificity of cytotoxic effects and potential off-target effects, 53L10 was also tested on CHO-K1 cells, stably transfected with DNA encoding 5T4 or PD-L1 to obtain cells expressing different levels of the two targets. Briefly, the cells were treated with increasing concentrations of 53L10, in co-cultures with human pre-activated lymphocytes, and after 72 h of treatment, the release of LDH was evaluated. As shown in **Figures 2B, C**, 53L10 did not show any significant effect on cells expressing undetectable levels of 5T4, whereas it induced strong effects on those expressing 5T4 at increasing levels. As for the cells transfected only with PD-L1, smaller effects of 53L10 were observed only at high concentrations, thus suggesting that the expression of PD-L1 alone did not significantly affect by itself the cytotoxicity and its presence (without 5T4) on other non-target cells, such as CHO-K1, and could be not sufficient to induce off-target side effects.

### 3.2 *In vivo* mechanism of action of 53L10 and serum stability

To investigate the mechanism of action of the 53L10 tribody, we generated a humanized mouse xenograft (15) model using A-549 tumor cells, as described in Materials and methods. Once the average tumor volume reached 100 mm^3^, mice were pre-treated with anti-asialo GM1 pAb to deplete murine NK cells, then engrafted with activated hPBMCs. Afterward, they were treated with five doses of either 53L10 or the bi-specific 53P (200 μg/mouse). As a negative control, a separate group of mice was treated with only PBS (**Figure 3A**). On day 10, we measured tumor growth and found that 53P caused a modest reduction in tumor growth compared to PBS only (approximately 20% of growth inhibition), while 53L10 had a more pronounced effect, reducing the tumor growth of approximately 50% (**Figure 3B**). These results confirmed the stronger anti-tumor effects of 53L10 compared to those of 53P, as previously reported (8), even though here the treatments started later after the growth of tumor, differently from the previous study where they were started at time 0. Furthermore, this novel *in vivo* experiment was not prolonged up to 50 days [to avoid repetition of already published data (8)], as it was designed with a different aim of clarifying the mechanism of action for its anti-tumor activity; thus, shorter time intervals were considered to be more appropriate. In **Table 2**, we have summarized the comparison of the tumor models and treatment schedules between this *in vivo* experiment and that previously reported (8) to better clarify the differences.

**Figure 3:**
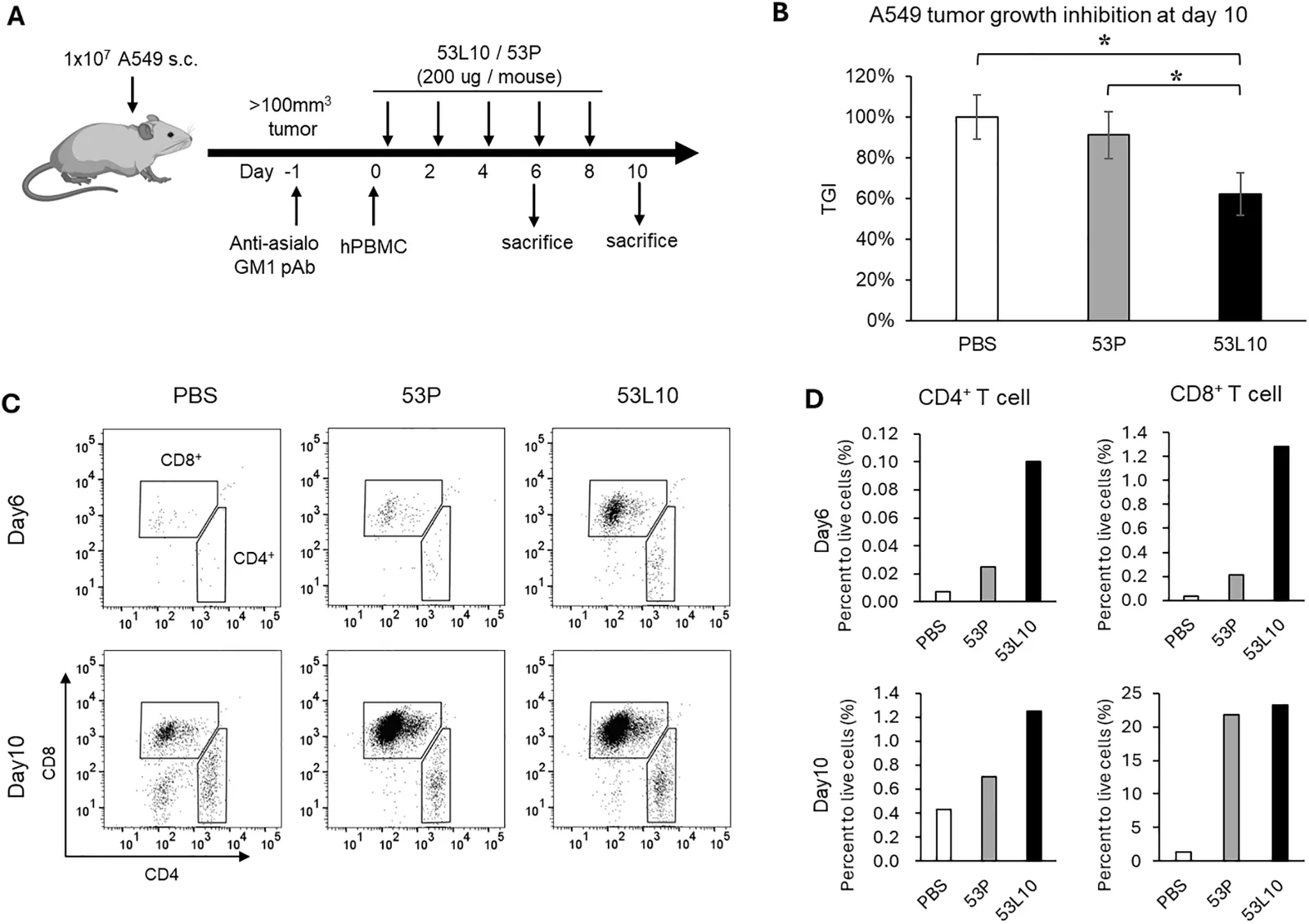
Analysis of tumor-infiltrating T cells in a PBMC-humanized A-549 xenograft model. **(A)** A-549 cells were transplanted s.c. into NOD-scid mice. When the average tumor volume reached 100 mm³, mice were treated with anti-sialo GM1 pAb. The next day, human PBMCs were introduced intraperitoneally, and 200 µg of 53P or 53L10 was subsequently administered intravenously once every 2 days. Tumors were resected on days 6 and 10. **(B)** Average tumor size on day 10 (mean of five mice per group). Error bars indicate standard deviations. Statistical significance was assessed by one-way ANOVA followed by Tukey’s *post-hoc* test and established as *p < 0.01. **(C, D)** On days 6 and 10, tumors from three mice per group were collected and pooled, and infiltrating lymphocytes were extracted. Human CD45^+^ lymphocytes were analyzed for CD4 and CD8 expression by flow cytometry.

**Table 2:** Comparison of the antibody constructs, tumor models, PBMC conditions, treatment schedules, endpoints, and principal findings of the previous and current studies.

Study	Passariello et al. (8)	Current study
Figure #	Figures 11 and 12	Figure 3
Antibody constructs	Tb535H (5T4/CD3 bi-specific) 53L1 (5T4/CD3/PD-L1 tri-specific) 53L10 (5T4/CD3/PD-L1 tri-specific) 53P (5T4/CD3 bi-specific) 53D (5T4/CD3/PD-1 tri-specific) 53G (5T4/CD3/LAG-3 tri-specific)	53P (5T4/CD3 bi-specific) 53L10 (5T4/CD3/PD-L1 tri-specific)
Tumor models	Mixture of A-549 and activated human PBMC was transplanted subcutaneously to NOD-scid mice at the same time	A-549 was transplanted s.c. into NOD-scid mice. When A-549 tumor reached ~100 mm³, human PBMCs were injected intraperitoneally.
PBMC conditions	Pre-activated by anti-CD3/anti-CD28 beads for 4 days before transplant	Pre-activated by anti-CD3/anti-CD28 beads for 3 days before transplant
Treatment schedules	Days 0, 2, 4, 6, 8, and 10. (day 0 = the day of A-549 transplantation)	Tribodies: Days 0, 2, 4, 6, and 8 (day 0 = the day of PBMC injection)
Endpoints	Tumor volume	Tumor volume on day 10 T-cell infiltration in tumors on days 6 and 10.
Principal findings	Tri-specific TCE tribodies having a checkpoint inhibitory arm showed superior in vivo anti-tumor activity compared to bi-specific TCE.	Treatment by 53L10 induced T-cell infiltration into A-549 tumor, more rapidly compared to 53P. Inhibition of PD-L1 enhanced T-cell infiltration.

Thus, we analyzed TILs from sacrificed mice at two key time points: day 6 and day 10. Flow cytometry analysis revealed that mice treated with 53L10 had a clear increase in both CD45^+^/CD4^+^ and CD45^+^/CD8^+^ T cells on fragmented tumor tissues compared to the PBS and 53P groups (**Figure 3C**). Specifically, in the 53L10 group, the percentage of live CD45^+^/CD4^+^ and CD45^+^/CD8^+^ T cells increased up to 1.2% on day 6 and 20% on day 10, whereas 53P only induced delayed increase after 10 days (**Figure 3D**). Overall, these results show that 53L10 induces an earlier and more efficient infiltration of effector T cells in the tumor microenvironment.

Since the stability of drugs at 37 °C is a critical factor for their therapeutic effects, we tested the stability of the novel tribodies in human serum at 37 °C for up to 72 h by monitoring their integrity and binding abilities. After incubation in serum for 24, 48, or 72 h at 37 °C, the percentage of undegraded protein was determined by Western blotting (**Figure 4B**) with an anti-His tag antibody. By using a Phosphorimager, the intensity of the electrophoretic bands obtained was measured and the percentage of undegraded tribodies was 100%. No degradation products with a lower molecular weight (MW) were observed.

**Figure 4:**
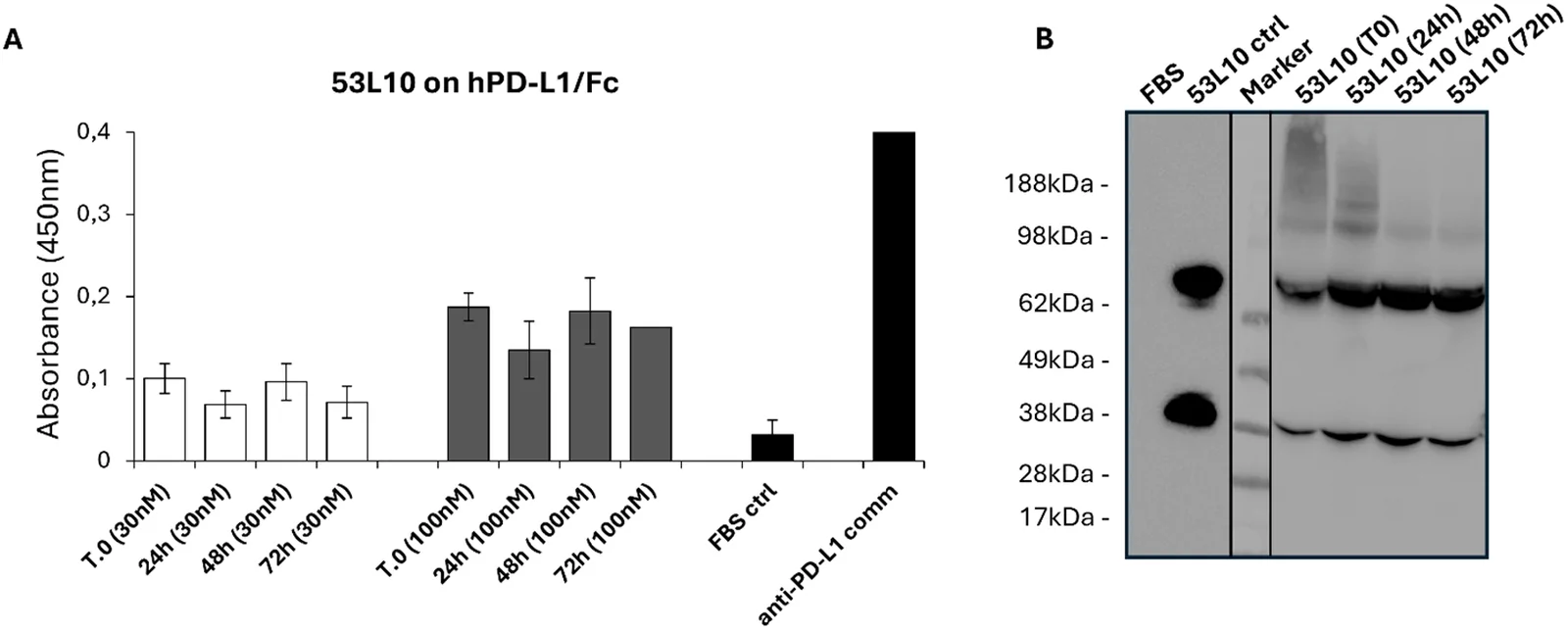
Serum stability of 53L10 tested by ELISA and Western blotting analysis. **(A)** 53L10 was incubated in 100% FBS at 37 °C for 24, 48, and 72 h and then analyzed for its functional binding by ELISAs on the immobilized hPD-L1/Fc protein. The untreated or treated 53L10 tribody was tested at concentrations of 30 nM (white bars) and 100 nM (gray bars), and the signals were detected by using the HRP-conjugated anti-His tag antibody. FBS or anti-PD-L1 commercial Ab was used as negative or positive control, respectively. The absorbance values at 450 nm were obtained by at least three independent experiments; error bars depicted means ± SD. **(B)** The integrity and stability of 53L10 after incubation in serum were analyzed by Western blotting analysis under non-reducing conditions by using the HRP-conjugated anti-His tag antibody.

The binding properties of the tribodies after incubation in the serum at different time intervals were assessed by ELISA on rPD-L1 immobilized protein. As shown in **Figure 4A**, the tribody incubated in human serum retained a binding affinity superimposable with that measured for the samples before incubation (*t* = 0), thus suggesting that 53L10 retains both its structural and functional properties over time.

## 4 Discussion

Novel tri-specific immunomodulatory tribodies (TRB) containing three different binding arms, i.e., the anti-TAA, anti-CD3, and anti-IC moieties, were recently generated in our laboratory, and one of them, named 53L10, including an anti-5T4 Fab and two scFvs specific for CD3 and PD-L1, respectively, showed the most potent *in vitro* effects on breast and lung cancer cells and led to tumor regression *in vivo* in a mouse model (11, 8).

Here, we further investigated the anti-tumor potential of the novel tri-specific 53L10 tribody by extending the study to a panel of tumor cell types with a more aggressive profile, including gastric, pancreatic, prostate, ovarian, and epidermoid tumor cells, which had not been tested before. Once confirmed by ELISAs that these cells express detectable levels of 5T4 and PD-L1 on the surface (16), we tested the cytotoxic effects of the 53L10 tribody on co-cultures of the mentioned new cells with hPBMCs demonstrating that tumor cell lysis induced by 53L10 was correlated with the expression levels of 5T4 and PD-L1. In particular, 53L10 induced a stronger anti-tumor response not only on lung and breast cancer cells already found to be sensitive in previous studies (8), but also on the highly aggressive gastric NCI-N87 and pancreatic PANC-1 cancer cells expressing high levels of PD-L1 (17, 18). No significant effects were observed on PD-L1-negative MCF7 cells or on transfected 5T4-negative CHO-K1 cells expressing only PD-L1, thus suggesting that both 5T4 and PD-L1 are required for the full efficacy of 53L10 and limited side effects could be expected on other adult normal tissues lacking 5T4 expression. The high activation of hPBMCs against cancer cells induced by 53L10, confirmed by cluster formation and the significant release of IL-2 and IFNγ (19, 20) in the supernatant, was much stronger than that of 53P, lacking the anti-PD-L1 arm. Furthermore, 53L10 showed a more potent anti-tumor efficacy *in vivo* than the bi-specific 53P, confirming the results of previous *in vivo* studies conducted for longer time intervals (11). In this work, we examined the effects after a few days post-administration to elucidate the biological mechanism of the anti-tumor activity of the novel TRB. In particular, we analyzed tumor-infiltrating lymphocytes in new models of humanized xenografts treated with either bi-specific (53P) or tri-specific (53L10) TRB, and found that both CD4^+^ and CD8^+^ T cells were increased at higher levels and earlier (6 days) in mice treated with 53L10 compared to mice treated with 53P.

Finally, we evaluated the stability of the 53L10 tribody in human serum and we found that it maintains both its structural integrity and functional biological activity. These findings suggest that the tribody could exhibit an extended half-life *in vivo*, potentially translating into prolonged therapeutic efficacy and a reduced administration frequency for patients. Thus, therapeutic effects could be prolonged post-administration, offering a better pharmacokinetic profile than the current BiTes in clinical use (21, 22). Moreover, the therapeutic potential of this novel tri-specific tribody is also supported by the encouraging combinatorial treatments of clinically validated bi-specifics with anti-PD-L1 mAbs such as those of clinical trials currently ongoing: NP39488 trial (23; NCT03533283) in phase Ib/II evaluating the combination of the bi-specific glofitamab (CD20 × CD3) with atezolizumab (anti-PD-L1) for the treatment of B-cell lymphoma; ERY974 trial (24; ClinicalTrials.gov, 2024; NCT05022927) in phase 1 evaluating the bi-specific ERY974 (GPC3 × CD3) in combination with atezolizumab or Tocilizumab for advanced solid tumors and hepatocellular carcinoma; and DeLLphi-305 (25**; NCT06211036**) in phase 3 examining Tarlatamab (a T-cell engager targeting both CD3 and DLL3 in tumor cells) in combination with Durvalumab (anti-PD-L1) for small cell lung cancer following previous encouraging results of DeLLphi-303 phase 1b evaluating the same combination.

Overall, these findings provide important evidence that the 53L10 T-cell engager tri-specific tribody could represent a valid therapeutic tool to induce T-cell infiltration and overcome tumor-induced immunosuppression also in aggressive types of tumors (26), thus supporting further preclinical evaluation of 53L10 as a new tool for multiple solid tumor-targeted therapeutic approaches.

## Data Availability

The raw data supporting the conclusions of this article will be made available by the authors, without undue reservation.
