# Supplementary material for: 53L10 T-cell engager tribody for immunotherapy of multiple solid tumors
Source: Front Immunol. 2026 Oct 6;17:1934039. doi: 10.3389/fimmu.2026.1934039 (PMC13680437; doi:10.3389/fimmu.2026.1934039)
Supplement: Supplementary file 1 [file DataSheet1.docx]

BRIEF RESEARCH REPORT ARTICLE

Section: Anticancer drugs

Title: 53L10 T cell engager tribody for immunotherapy of multiple solid tumors

**Margherita Passariello^1,2±^_,_ Martina Belardo^1,2±^_,_ Toshikazu Inoue^3^, Kentaro Kajiwara^3^, Rosa Rapuano Lembo^2,4^, Shu-ichi Hashimoto^3*^ and Claudia De Lorenzo^1,2*^**

***Supplementary Material***

Supplementary Figures

**Supplementary Figure 1. IL-2 and IFNγ released in co-cultures of tumor cells with hPBMCs treated with 53L10 or 53P.** The supernatants of the indicated tumor cells, co-cultured with hPBMCs (5:1 effector: target ratio) and treated with 53L10 (**A**-**B**) or 53P (**C**-**D**), used at increasing concentrations for 48 hours, were tested by using the cytokine secretion kits (from R&D systems) for measuring the levels of IL-2 and IFNγ. The values reported were expressed in pg/mL as the mean of determinations obtained in three independent experiments. Error bars depicted means ± SD.
